# Supplementary material for: Understanding risk factors for disordered eating symptomatology in athletes: A prospective study
Source: PLoS One. 2021 Sep 24;16(9):e0257577. doi: 10.1371/journal.pone.0257577 (PMC8462695; doi:10.1371/journal.pone.0257577)
Supplement: Attachment — Submitted filename: PONE-D-21-04001 responses to reviewers.docx [file pone.0257577.s002.docx]

PONE-D-21-04001

Please include the following items when submitting your revised manuscript:

- A rebuttal letter that responds to each point raised by the academic editor and reviewer(s). This letter should be uploaded as separate file and labeled 'Response to Reviewers'.
- A marked-up copy of your manuscript that highlights changes made to the original version. This file should be uploaded as separate file and labeled 'Revised Manuscript with Track Changes'.
- An unmarked version of your revised paper without tracked changes. This file should be uploaded as separate file and labeled 'Manuscript'.

2. Please provide additional details regarding participant consent. In the ethics statement in the Methods and online submission information, please ensure that you have specified what type you obtained (for instance, written or verbal, and if verbal, how it was documented and witnessed). If your study included minors, state whether you obtained consent from parents or guardians. If the need for consent was waived by the ethics committee, please include this information.

*Further detail of the consent process has been provided in the Method section and in the online submission.*

3.  Thank you for stating the following financial disclosure:

"NO-The funders had no role in the study design, data collection and analysis, decision to publish, or preparation of the manuscript. "

At this time, please address the following queries:

a. Please clarify the sources of funding (financial or material support) for your study. List the grants or organizations that supported your study, including funding received from your institution.

*No funding was provided for the study from any grants or organizations. The first author was a self-funded PhD student who provided the renumeration to participants from their own funds.*

b. State what role the funders took in the study. If the funders had no role in your study, please state: “The funders had no role in study design, data collection and analysis, decision to publish, or preparation of the manuscript.”

*n/a*

c. If any authors received a salary from any of your funders, please state which authors and which funders.

*n/a*

d. If you did not receive any funding for this study, please state: “The authors received no specific funding for this work.”

Please include your amended statements within your cover letter; we will change the online submission form on your behalf.

4. We noted in your submission details that a portion of your manuscript may have been presented or published elsewhere.

"Yes, figure 1 and figure 2 have been published before and are needed as reference for clarity in the manuscript. They are appropriately cited and accredited to authors"

Please clarify whether this publication was peer-reviewed and formally published. If this work was previously peer-reviewed and published, in the cover letter please provide the reason that this work does not constitute dual publication and should be included in the current manuscript.

*Reviewer 1 suggested that we remove Figure 1 from the manuscript so we have done this. Figure 2 (now figure 1) was previously published in a peer-reviewed journal, but has been included as this model as it was developed by the current research group and is required in order to explain the research questions in the current study. It has also been appropriately cited.*

5.  We note that you have indicated that data from this study are available upon request. PLOS only allows data to be available upon request if there are legal or ethical restrictions on sharing data publicly. For more information on unacceptable data access restrictions, please see http://journals.plos.org/plosone/s/data-availability#loc-unacceptable-data-access-restrictions.

In your revised cover letter, please address the following prompts:

a) If there are ethical or legal restrictions on sharing a de-identified data set, please explain them in detail (e.g., data contain potentially sensitive information, data are owned by a third-party organization, etc.) and who has imposed them (e.g., an ethics committee). Please also provide contact information for a data access committee, ethics committee, or other institutional body to which data requests may be sent.

b) If there are no restrictions, please upload the minimal anonymized data set necessary to replicate your study findings as either Supporting Information files or to a stable, public repository and provide us with the relevant URLs, DOIs, or accession numbers. For a list of acceptable repositories, please see http://journals.plos.org/plosone/s/data-availability#loc-recommended-repositories.

We will update your Data Availability statement on your behalf to reflect the information you provide.

*We have uploaded the de-identified data set*

Reviewer #1:

1. Introduction: I suggest removing figure 1 and moving figure 2 (and text) in the method section.

*Thank you for these suggestions. We have removed figure 1 and renumbered the remaining figures. We have carefully considered whether it is possible to move figure 2 (now re-numbered as figure 1) to the Method, however it does not seem possible to do this because the explanation of the role of the T1 model leads to the hypotheses for the current study. As such this needs to be introduced in the introduction section.*

2. Methods: Please clarify better (possible with references) this sentences: “The inclusion criteria at T1 were that participants had to be over the age of 18; had to consider themselves to be an athlete (determined with a simple yes/no answer to the question “Do you identify as an athlete?”); had to be training for a minimum of ten hours a week; and had to be actively competing. These criteria were set such that those included in the study were athletes, rather than just regular exercisers.”

*We have revised the text as follows to clarify inclusion criteria:*

*Those who identified themselves as athletes and performed at least ten hours of training per week and compete in their current sport were invited to participate. These inclusion criteria were used to determine that those completing the questionnaire were athletes rather than zealous exercisers. Self-identification as an athlete was the primary criterion for inclusion with actively competing also acting as an important determinant of inclusion. As a final back-up, a minimum number of ten hours of participation was set based on the expertise of the first author.*

3. I suggest moving volunteers’ characteristics and Table 1 in the results section.

*Thank you, this has been done*

4. Please add the strobe statement for observational studies in PLOS Medicine

STROBE Statement: Observational Studies: Getting clear about transparency ([strobe-statement.org](http://strobe-statement.org))

*We have examined in detail the various STROBE checklists but there do not appear to be any that are appropriate for a prospective study.*

Reviewer #2: 
1. The paper may have a certain implication, however it is really difficult to read. I advise authors to shorten and focus it.

*The paper has been extensively revised to improve readability and considerably shortened*

2. The abstract should be better presented I including more numerical results

*The abstract has been revised and restructured to make it more concise.*

3. The Introduction section is too long, and need to be shortened not to exceed 1 ½ page.

*The introduction has been reduced to 1 and a half pages as suggested.*

4. The Result section is dispersive and should be more focused.

*Efforts have been made to clarify and simplify the results section somewhat. However, it has been difficult to cut much material due to the complex nature of the analysis. We do hope the reviewers agree that it is much improved.*

5. The quality of the tables and figures should be improved

*Many of the tables have been redesigned to improve layout and readability.*
